# Interinstitutional analysis of the outcome after surgery for type A aortic dissection

**DOI:** 10.1007/s00068-023-02248-2

**Published:** 2023-02-24

**Authors:** Fausto Biancari, Angelo M. Dell’Aquila, Giuseppe Gatti, Andrea Perrotti, Amélie Hervé, Joseph Touma, Matteo Pettinari, Sven Peterss, Joscha Buech, Konrad Wisniewski, Tatu Juvonen, Mikko Jormalainen, Caius Mustonen, Andreas Rukosujew, Till Demal, Lenard Conradi, Marek Pol, Petr Kacer, Francesco Onorati, Cecilia Rossetti, Igor Vendramin, Daniela Piani, Mauro Rinaldi, Luisa Ferrante, Eduard Quintana, Robert Pruna-Guillen, Javier Rodriguez Lega, Angel G. Pinto, Metesh Acharya, Zein El-Dean, Mark Field, Amer Harky, Manoj Kuduvalli, Francesco Nappi, Sebastien Gerelli, Dario Di Perna, Enzo Mazzaro, Stefano Rosato, Antonio Fiore, Giovanni Mariscalco

**Affiliations:** 1 Heart and Lung Center, Helsinki University Hospital, University of Helsinki, 00029 Helsinki, Finland; 2 Department of Medicine, South-Karelia Central Hospital, University of Helsinki, Lappeenranta, Finland; 3 Department of Cardiothoracic Surgery, University Hospital Muenster, Muenster, Germany; 4 Division of Cardiac Surgery, Cardio-Thoracic and Vascular Department, Azienda Sanitaria Universitaria Giuliano Isontina, Trieste, Italy; 5 Department of Thoracic and Cardiovascular Surgery, University of Franche-Comte, Besancon, France; 6 Department of Vascular Surgery, Hôpitaux Universitaires Henri Mondor, Assistance Publique-Hôpitaux de Paris, Creteil, France; 7 Department of Cardiac Surgery, Ziekenhuis Oost Limburg, Genk, Belgium; 8 LMU University Hospital, Ludwig Maximilian University, Munich, Germany; 9 German Centre for Cardiovascular Research, Partner Site Munich Heart Alliance, Munich, Germany; 10 Anesthesia and Critical Care, Research Unit of Surgery, University of Oulu, Oulu, Finland; 11 Department of Cardiovascular Surgery, University Heart and Vascular Center Hamburg, Hamburg, Germany; 12 Department of Cardiac Surgery, Third Faculty of Medicine, Charles University and University Hospital Kralovske Vinohrady, Prague, Czech Republic; 13 Division of Cardiac Surgery, University of Verona Medical School, Verona, Italy; 14 Cardiothoracic Department, University Hospital, Udine, Italy; 15 Cardiac Surgery, Molinette Hospital, University of Turin, Turin, Italy; 16 Department of Cardiovascular Surgery, Hospital Clínic de Barcelona, University of Barcelona, Barcelona, Spain; 17 Cardiovascular Surgery Department, University Hospital Gregorio Marañón, Madrid, Spain; 18 Department of Cardiac Surgery, Glenfield Hospital, Leicester, UK; 19 Liverpool Centre for Cardiovascular Sciences, Liverpool Heart and Chest Hospital, Liverpool, UK; 20 Department of Cardiac Surgery, Centre Cardiologique du Nord de Saint-Denis, Paris, France; 21 Centre Hospitalier Annecy Genevois, Épagny-Metz-Tessy, France; 22 Center for Global Health, National Health Institute, Rome, Italy; 23 Department of Cardiac Surgery, Hôpitaux Universitaires Henri Mondor, Assistance Publique-Hôpitaux de Paris, Creteil, France

**Keywords:** Type A aortic dissection, Aortic dissection, Volume

## Abstract

**Purpose:**

To evaluate the impact of individual institutions on the outcome after surgery for Stanford type A aortic dissection (TAAD).

**Methods:**

This is an observational, multicenter, retrospective cohort study including 3902 patients who underwent surgery for TAAD at 18 university and non-university hospitals.

**Results:**

Logistic regression showed that four hospitals had increased risk of in-hospital mortality, while two hospitals were associated with decreased risk of in-hospital mortality. Risk-adjusted in-hospital mortality rates were lower in four hospitals and higher in other four hospitals compared to the overall in-hospital mortality rate (17.7%). Participating hospitals were classified as overperforming or underperforming if their risk-adjusted in-hospital mortality rate was lower or higher than the in-hospital mortality rate of the overall series, respectively. Propensity score matching yielded 1729 pairs of patients operated at over- or underperforming hospitals. Overperforming hospitals had a significantly lower in-hospital mortality (12.8% vs. 22.2%, *p* < 0.0001) along with decreased rate of stroke and/or global brain ischemia (16.5% vs. 19.9%, *p* = 0.009) compared to underperforming hospitals. Aggregate data meta-regression of the results of participating hospitals showed that hospital volume was inversely associated with in-hospital mortality (*p* = 0.043). Hospitals with an annual volume of less than 15 cases had an increased risk of in-hospital mortality (adjusted OR, 1.345, 95% CI 1.126–1.607).

**Conclusion:**

The present findings indicate that there are significant differences between hospitals in terms of early outcome after surgery for TAAD. Low hospital volume may be a determinant of poor outcome of TAAD.

**Trial registration:**

ClinicalTrials.gov Identifier: NCT04831073.

## Introduction

Acute Stanford type A aortic dissection (TAAD) is associated with high mortality [1]. Emergency surgical repair of TAAD is associated with early mortality rates higher than 10% and significant cerebral and visceral complications [2]. Interinstitutional differences may exist in terms of early and late outcome after surgery for TAAD, but only a few studies have evaluated this issue. Since, centralization of aortic surgery is advocated as a measure to improve the results of aortic surgery [3], more data are needed to support the creation of centers dedicated to the surgical and endovascular treatment of aortic diseases. We performed a multicenter, observational study on the outcome after surgery for acute TAAD and we hypothesized that differences in the outcome might exist between the participating centers. We evaluated this issue in the present analysis.

## Methods

### Study design

The present is an observational, multicenter, retrospective cohort study, which was approved by the Ethical Review Board of the Helsinki University Hospital, Finland (April 21, 2021, diary no. HUS/237/2021) and by the Ethical Review Board of each participating hospital. The requirement for informed consent was waived because of the retrospective nature of this study. The European Registry of Type A Aortic Dissection (ERTAAD) included consecutive patients who underwent surgery for acute TAAD at 18 centers of cardiac surgery located in eight European countries (Table 1) from January 1, 2005 to March 31, 2021. Data were retrospectively collected into a Microsoft Access datasheet (Redmond, Washington, USA) with pre-specified baseline, operative and outcome variables. Data on the date of death and repeated aortic intervention were collected retrospectively from electronic national registries as well as by contacting regional hospitals, patients and their relatives. Completeness of follow-up data varied significantly between centers and this was due to country-specific availability of methods to get data on follow-up.

**Table 1:** Patients and their in-hospital and 10-year all-cause mortality in the participating hospitals

No	Participating hospitals	No. of patients (%)	Study period	In-hospital mortality (%)	10-year mortality (%)
1	A	132 (3.4)	2011–2021	18.9	41.4
2	B	156 (4.0)	2009–2021	16.7	46.3
3	C	249 (6.4)	2005–2021	18.5	46.5
4	D	69 (1.8)	2007–2021	27.5	45.9
5	E	281 (7.2)	2010–2021	17.1	37.9
6	F	329 (8.4)	2005–2021	13.7	38.3
7	G	133 (3.4)	2005–2021	19.4	54.6
8	H	172 (4.4)	2008–2021	11.6	53.1
9	I	105 (2.7)	2005–2021	27.6	47.6
10	L	341 (8.7)	2005–2021	12.9	34.5
11	M	308 (7.9)	2005–2021	17.9	52.2
12	N	167 (4.3)	2007–2021	25.1	44.3
13	O	293 (7.5)	2006–2021	27.0	44.3
14	P	81 (2.1)	2010–2021	30.9	75.4
15	Q	492 (12.6)	2005–2021	18.9	51.8
16	R	141 (3.6)	2005–2021	17.7	51.9
17	S	182 (4.7)	2010–2021	9.9	54.3
18	T	271 (6.9)	2008–2021	8.9	57.2

### Study participants

The study participants were recruited according to the following inclusion criteria: (1) TAAD or intramural hematoma involving the ascending aorta; (2) patients aged > 18 years; (3) symptoms started within 7 days prior to surgery; (4) primary surgical repair of acute TAAD; (5) any other major cardiac surgical procedure concomitant with surgery for TAAD [4]. The exclusion criteria were the following: (1) patients aged < 18 years; (2) onset of symptoms > 7 days prior to surgery; (3) prior procedure for TAAD; (4) retrograde TAAD (with primary tear located in the descending aorta); (5) concomitant endocarditis; (6) TAAD secondary to blunt or penetrating chest trauma [4].

Information regarding the definition criteria of risk factors have been previously reported [4]. Surgeon was defined experienced if had performed at least 20 elective or urgent procedures on the ascending aorta/aortic arch in the preceding year.

### Outcome measures

The primary outcome of this study was in-hospital mortality, i.e., all-cause death occurred during the index hospitalization. The secondary outcomes were stroke, global brain ischemia, as well as a composite end-point including in-hospital mortality, stroke and global brain ischemia. Other secondary outcomes were need of mechanical circulatory support, dialysis, reoperation for intrathoracic bleeding, tracheostomy, mesenteric ischemia, 10-year rates of mortality as well as distal and proximal aortic reoperations. Definition criteria for these outcomes have been previously reported [4].

### Statistical analysis

Categorical variables are reported as counts and percentages. Continuous variables are reported as means and standard deviations. Univariate analysis of continuous variables was performed using the Kruskal–Wallis’ test and of categorical variables using the Chi-square test. Survival analysis was performed using the Kaplan–Meier and competing risk analysis methods. Competing risk analyses using the Fine-Gray test with all-cause death as a competing event were performed to estimate difference between study groups in cumulative incidence of aortic reoperations. Logistic regression using the stepwise backward method was performed with the in-hospital mortality as the dependent variables and considering participating hospitals along with the following covariates associated with increased risk of in-hospital mortality: age, iatrogenic TAAD, preoperative cardiac massage, cerebral malperfusion, mesenteric malperfusion, peripheral malperfusion, aortic root replacement and aortic arch replacement and procedure performed by an experienced aortic surgeon. The risk-adjusted rate of in-hospital mortality was calculated by dividing, for each participating centre, the observed number of events by the expected number of events, and by multiplying this ratio by the average event rate of the entire series. The expected numbers of events were estimated using logistic regression. After plotting the risk-adjusted rates, participating hospitals were classified either as underperforming or overperforming if their risk-adjusted rate was higher or lower than the in-hospital mortality of the overall series, respectively. Considering the expected imbalance in the baseline and operative covariates, a propensity score matching analysis was performed employing a caliper width of 0.2 the standard deviation of the logit. Propensity score was calculated with logistic regression considering over- and under-performing hospitals as dependent variable and including all the covariates listed in Table 2, with the exception of aortic cross-clamping time and cardiopulmonary bypass time because they were expected to be part of the participating hospital’s characteristics. A standardized difference < 0.10 was considered as an acceptable balance between covariates of the study groups. The prognostic impact of hospital annual volume on in-hospital mortality was estimated using aggregated data meta-regression with random-effects. Furthermore, the median hospital volume of the participating hospitals was considered as a cutoff for high and low-volume hospitals and its effect was adjusted in multivariable logistic regression. Statistical analyses were performed with the SPSS (version 27.0, SPSS Inc., IBM, Chicago, Illinois, USA), Stata (version 15.1, StataCorp LLC, College Station, Texas, USA) and Open meta-analyst (version 2014, CESH, Brown University, Rhode Island, USA, cebm.brown.edu/openmeta/) statistical softwares.

**Table 2:** Patients’ characteristics and operative data of patients operated in the participating centers

Characteristics	Centers									
	Overall *N* = 3902	A *N* = 132	B *N* = 156	C *N* = 249	D *D* = 69	E *E* = 281	F *N* = 329	G *N* = 133	H *N* = 172	I *N* = 105
Age, mean (SD), y	63.3 (13.0)	63.6 (14.2)	61.8 (11.6)	63.1 (13.6)	62.9 (12.4)	62.8 (13.4)	61.4 (13.2)	62.1 (14.0)	59.3 (13.2)	61.4 (12.6)
Females, No. (%)	2717 (69.6)	42 (31.8)	47 (30.1)	72 (28.9)	29 (42.0)	83 (29.5)	110 (33.4)	39 (29.3)	54 (31.4)	31 (29.5)
Genetic syndrome, No. (%)	81 (2.1)	4 (3.0)	9 (5.8)	4 (1.6)	1 (1.5)	8 (2.9)	6 (1.8)	4 (3.0)	11 (6.4)	2 (1.9)
Bicuspid aortic valve, No. (%)	151 (3.9)	5 (3.8)	6 (3.9)	20 (8.0)	4 (5.8)	14 (5.0)	25 (7.6)	7 (5.3)	7 (4.1)	9 (8.7)
Iatrogenic dissection, No. (%)	103 (2.6)	1 (0.8)	5 (3.2)	6 (2.4)	5 (7.3)	15 (5.3)	7 (2.1)	4 (3.0)	7 (4.1)	4 (3.8)
Diabetes, No. (%)	196 (5.0)	4 (3.0)	15 (9.6)	15 (6.0)	3 (4.4)	14 (5.0)	23 (7.0)	4 (3.0)	9 (5.2)	1 (0.9)
Stroke, No. (%)	234 (6.0)	4 (3.0)	9 (5.8)	13 (5.2)	3 (4.5)	19 (6.8)	19 (5.8)	2 (1.5)	3 (1.7)	4 (3.8)
Pulmonary disease, No. (%)	327 (8.4)	16 (12.1)	17 (10.9)	20 (8.0)	7 (10.1)	29 (10.3)	29 (8.8)	6 (4.5)	23 (13.4)	6 (5.7)
Extracardiac arteriopathy, No. (%)	199 (5.1)	8 (6.1)	12 (7.7)	10 (4.0)	8 (11.6)	9 (3.2)	13 (4.0)	5 (3.8)	6 (3.5)	2 (1.9)
Prior cardiac surgery, No. (%)	88 (2.3)	3 (2.3)	6 (3.9)	6 (2.4)	2 (2.9)	9 (3.2)	11 (3.3)	2 (1.5)	10 (5.8)	2 (1.9)
Cardiac massage, No. (%)	163 (4.2)	0 (0)	5 (3.2)	10 (4.0)	4 (5.8)	11 (3.9)	19 (5.8)	10 (7.5)	10 (5.8)	4 (5.8)
Shock requiring inotropes, No. (%)	638 (16.4)	25 (18.9)	39 (25.0)	54 (21.7)	6 (8.7)	20 (7.1)	86 (26.1)	9 (6.8)	19 (11.1)	8 (11.1)
Cerebral malperfusion, No. (%)	838 (21.5)	45 (34.1)	30 (19.2)	76 (30.5)	11 (15.9)	62 (22.1)	50 (15.2)	21 (15.8)	16 (9.3)	25 (23.8)
Spinal malperfusion, No. (%)	83 (2.1)	8 (6.1)	9 (5.9)	0 (0)	1 (1.5)	8 (2.9)	11 (3.3)	1 (08)	0 (0)	8 (7.6)
Renal malperfusion, No. (%)	364 (9.3)	22 (16.7)	32 (20.5)	19 (7.6)	0 (0)	40 (14.2)	24 (7.8)	14 (10.5)	22 (12.8)	4 (3.8)
Mesenteric malperfusion, No. (%)	162 (4.2)	18 (13.6)	10 (6.4)	13 (5.2)	1 (1.5)	16 (5.7)	10 (3.0)	6 (4.5)	4 (2.3)	3 (2.9)
Peripheral malperfusion, No. (%)	562 (14.4)	25 (18.9)	42 (26.9)	38 (15.3)	10 (14.5)	40 (14.2)	67 (20.3)	20 (15.4)	20 (11.6)	21 (20.0)
DeBakey type I dissection, No. (%)	3275 (83.9)	113 (85.6)	139 (89.1)	221 (88.8)	66 (95.7)	249 (88.6)	286 (86.9)	117 (87.8)	136 (79.1)	86 (81.9)
Operative data										
Experienced aortic surgeon, No. (%)	3229 (82.8)	131 (99.2)	153 (98.1)	231 (92.8)	33 (47.8)	155 (55.2)	128 (38.9)	111 (83.5)	169 (98.3)	105 (100)
Partial or total arch repair, No. (%)	776 (19.9)	21 (15.9)	69 (44.2)	14 (5.6)	20 (29.0)	73 (26.0)	32 (9.7)	20 (15.0)	30 (17.4)	12 (11.4)
Aortic root repair, No. (%)	1097 (28.1)	59 (44.7)	35 (22.4)	88 (35.3)	37 (53.6)	64 (22.8)	95 (28.9)	33 (24.8)	129 (75.0)	39 (37.1)
XCT time, mean (SD), min	120 (59)	103 (44)	106 (48)	102 (45)	184 (71)	116 (72)	104 (40)	119 (48)	201 (76)	119 (47)
CPB time, mean (SD), min	216 (89)	144 (58)	198 (69)	194 (81)	274 (101)	245 (94)	204 (61)	209 (66)	349 (110)	200 (67)

Characteristics	Centers									
	L *N* = 341	M *N* = 308	N *N* = 167	O *N* = 293	P *N* = 81	Q *N* = 492	R *N* = 141	S *N* = 182	T *T* = 271	*P* value
Age, mean (SD), y	62.8 (13.3)	62.9 (13.0)	63.2 (13.3)	63.8 (13.1)	64.1 (11.6)	65.4 (12.3)	65.5 (12.7)	65.2 (12.2)	65.2 (12.1)	0.0001
Females, No. (%)	115 (33.7)	93 (30.2)	45 (27.0)	96 (32.8)	26 (32.1)	134 (27.2)	39 (27.7)	49 (26.9)	81 (29.9)	0.664
Genetic syndrome, No. (%)	6 (1.7)	9 (2.9)	2 (1.0)	7 (2.4)	0 (0)	3 (0.6)	1 (0.7)	2 (1.1)	2 (0.7)	0.001
Bicuspid aortic valve, No. (%)	10 (2.9)	1 (0.3)	3 (1.8)	5 (1.7)	2 (2.5)	13 (2.6)	4 (2.8)	8 (4.4)	8 (3.0)	< 0.0001
Iatrogenic dissection, No. (%)	11 (3.2)	6 (2.0)	1 (0.6)	8 (2.7)	4 (4.9)	7 (1.4)	5 (3.6)	4 (2.2)	3 (1.1)	0.031
Diabetes, No. (%)	9 (2.6)	8 (2.6)	15 (9.0)	16 (5.5)	7 (8.6)	28 (5.7)	5 (3.6)	8 (4.4)	12 (4.4)	0.011
Stroke, No. (%)	8 (8.4)	22 (7.1)	8 (4.8)	6 (2.1)	5 (6.2)	20 (4.1)	0 (0)	4 (2.2)	4 (1.5)	0.001
Pulmonary disease, No. (%)	23 (6.7)	41 (13.1)	8 (4.8)	14 (4.8)	3 (3.7)	39 (7.9)	11 (7.8)	21 (11.5)	14 (5.2)	0.001
Extracardiac arteriopathy, No. (%)	11 (3.2)	63 (20.5)	1 (0.6)	4 (1.4)	2 (2.5)	23 (4.7)	16 (11.4)	3 (1.7)	3 (1.1)	< 0.0001
Prior cardiac surgery, No. (%)	2 (0.6)	12 (3.9)	4 (2.4)	13 (4.4)	4 (4.9)	13 (2.6)	4 (2.8)	7 (3.9)	12 (4.4)	0.285
Cardiac massage, No. (%)	11 (3.9)	19 (5.8)	8 (4.8)	15 (5.1)	8 (9.9)	15 (3.1)	2 (2.1)	9 (5.0)	5 (1.9)	0.015
Shock requiring inotropes, No. (%)	8 (7.6)	126 (40.9)	5 (3.0)	50 (17.1)	5 (6.2)	47 (9.6)	13 (9.3)	45 (24.7)	66 (24.4)	< 0.0001
Cerebral malperfusion, No. (%)	60 (17.6)	149 (48.4)	13 (7.8)	46 (15.7)	15 (18.5)	105 (21.3)	21 (14.9)	38 (20.9)	46 (17.0)	< 0.0001
Spinal malperfusion, No. (%)	5 (1.5)	8 (2.6)	7 (4.2)	4 (1.4)	0 (0)	8 (1.6)	1 (0.7)	3 (1.7)	0 (0)	< 0.0001
Renal malperfusion, No. (%)	34 (10.0)	17 (5.5)	2 (1.2)	40 (13.7)	9 (11.1)	27 (5.5)	19 (13.5)	17 (9.3)	22 (8.1)	< 0.0001
Mesenteric malperfusion, No. (%)	11 (3.2)	2 (0.7)	2 (1.2)	21 (7.2)	3 (3.7)	11 (2.2)	10 (7.1)	11 (6.0)	10 (3.7)	< 0.0001
Peripheral malperfusion, No. (%)	14 (4.1)	111 (36.0)	0 (0)	18 (6.1)	11 (13.6)	40 (8.1)	14 (9.9)	25 (13.7)	27 (10.0)	< 0.0001
DeBakey type I dissection, No. (%)	277 (81.2)	274 (88.9)	140 (83.8)	256 (87.4)	72 (88.9)	392 (79.7)	111 (78.7)	176 (96.7)	164 (60.5)	< 0.0001
Operative data										
Experienced aortic surgeon, No. (%)	341 (100)	272 (88.3)	162 (97.0)	289 (98.6)	79 (97.5)	444 (90.2)	120 (85.1)	106 (58.2)	200 (73.8)	< 0.0001
Partial or total arch repair, No. (%)	80 (23.5)	83 (27.0)	27 (16.2)	88 (30.0)	7 (8.6)	49 (10.0)	14 (9.9)	71 (39.1)	66 (24.4)	< 0.0001
Aortic root repair, No. (%)	139 (40.8)	67 (21.8)	19 (11.4)	84 (28.7)	22 (27.2)	69 (14.0)	26 (18.4)	28 (15.4)	64 (23.6)	
XCT time, mean (SD), min	130 (53)	116 (46)	70 (30.5)	140 (66)	115 (55)	112 (46)	124 (60)	117 (59)	126 (59)	0.0001
CPB time, mean (SD), min	211 (67)	214 (72)	113 (45)	231 (107)	237 (75)	207 (81)	228 (76)	218 (83)	234 (82)	0.0001

*CPB* cardiopulmonary bypass, *SD* standard deviation, *XCT* aortic cross clamping time

## Results

### Participant characteristics

The mean age of patients was 63.3 (13.0) years, and there were 1185 (30.4%) females. DeBakey type I dissection was present in 3275 (83.8%) patients. TAAD was of iatrogenic origin in 103 (2.6%) of patients. The proportion of patients in the participating hospitals is summarized in Table 1. Participating hospitals significantly differed in terms of patients’ characteristics and operative approach (Table 2). Early and 10-year outcome differed between participating hospitals as well (Table 3).

**Table 3:** Early and late outcomes of patients operated at the participating centers

Outcomes	Centers									
	Overall *N* = 3902	A *N* = 132	B *N* = 156	C *N* = 249	D *D* = 69	E *E* = 281	F *N* = 329	G *N* = 133	H *N* = 172	I *N* = 105
Early outcomes										
Hospital mortality, No. (%)	689 (17.7)	25 (18.9)	26 (16.7)	46 (18.5)	19 (27.4)	48 (17.1)	45 (13.7)	26 (19.6)	20 (11.6)	29 (27.6)
Stroke/global brain ischemia, No. (%)	723 (18.5)	20 (15.2)	19 (12.2)	69 (27.7)	9 (13.0)	68 (24.0)	85 (25.8)	25 (18.8)	38 (22.1)	20 (19.1)
Composite end-point, No. (%)	1152 (29.5)	35 (26.5)	38 (24.4)	97 (39.0)	22 (31.9)	98 (34.9)	109 (33.1)	40 (30.1)	51 (29.7)	38 (36.2)
Mesenteric ischemia, No. (%)	149 (3.8)	5 (3.8)	3 (1.9)	28 (11.2)	0 (0)	3 (1.1)	4 (1.2)	6 (4.5)	3 (1.7)	6 (5.8)
Dialysis, No. (%)	559 (14.3)	12 (9.1)	21 (13.5)	39 (15.7)	4 (5.8)	28 (10.0)	50 (15.2)	15 (11.3)	7 (4.1)	11 (10.7)
Reoperation for bleeding, No. (%)	549 (14.1)	18 (13.6)	40 (25.6)	50 (20.1)	5 (7.3)	27 (9.6)	39 (11.9)	23 (17.3)	12 (7.0)	12 (11.4)
MCS, No. (%)	141 (3.6)	0 (0)	1 (0.6)	12 (4.8)	2 (2.9)	13 (4.6)	6 (1.8)	6 (4.5)	2 (1.2)	4 (3.8)
10-year outcomes										
Mortality, (%)	47.8	41.4	46.3	46.5	45.9	37.9	38.3	55.6	53.1	47.7
Distal aortic reoperation, (%)	4.5	5.1	7.1	6.7	3.6	26.8	8.0	5.7	4.0	13.3
Proximal aortic reoperation, (%)	4.3	7.2	5.5	4.8	2.9	4.7	5.2	5.6	0	5.2
Lost to follow-up (%)	13.2	1.5	0.6	0.8	5.8	44.8	6.7	12.8	18.0	7.6

Outcomes	Centers									
	L *N* = 341	M *N* = 308	N *N* = 167	O *N* = 293	P *N* = 81	Q *N* = 492	R *N* = 141	S *N* = 182	T *T* = 271	*P* value
Early outcomes										
Hospital mortality, No. (%)	44 (12.9)	55 (17.9)	42 (25.2)	79 (27.0)	25 (30.9)	93 (18.9)	25 (17.7)	19 (9.9)	24 (8.9)	< 0.0001
Stroke/global brain ischemia, No. (%)	32 (9.4)	35 (11.4)	42 (25.2)	61 (20.8)	30 (37.0)	80 (16.3)	8 (5.7)	28 (15.4)	54 (19.9)	< 0.0001
Composite end-point, No. (%)	69 (20.2)	72 (23.3)	62 (37.1)	101 (34.4)	44 (54.3)	137 (27.9)	30 (21.28	39 (21.43)	70 (25.8)	< 0.0001
Mesenteric ischemia, No. (%)	6 (1.8)	23 (7.5)	6 (3.4)	14 (4.8)	4 (4.9)	15 (3.1)	9 (6.4)	9 (5.0)	5 (1.9)	< 0.0001
Dialysis, No. (%)	53 (15.5)	91 (29.6)	4 (2.4)	49 (16.7)	29 (35.8)	69 (14.0)	10 (7.1)	35 (19.2)	32 (11.8)	< 0.0001
Reoperation for bleeding, No. (%)	70 (20.5)	56 (18.8)	15 (9.0)	22 (7.5)	33 (40.7)	43 (8.7)	19 (13.5)	30 (16.5)	35 (12.9)	< 0.0001
MCS, No. (%)	16 (4.7)	21 (6.8)	4 (2.4)	31 (10.6)	2 (2.5)	9 (1.8)	0 (0)	4 (2.2)	8 (2.9)	< 0.0001
10-year outcomes										
Mortality, (%)	34.5	52.2	44.3	44.3	75.4	51.8	51.9	54.3	57.2	0.005
Distal aortic reoperation, (%)	8.3	10.4	1.0	4.5	12.1	3.7	2.1	10.1	6.4	< 0.0001
Proximal aortic reoperation, (%)	5.6	7.3	1.0	3.5	10.2	2.1	0	2.9	4.7	< 0.0001
Lost to follow-up (%)	22.3	4.5	11.4	30.7	6.2	16.7	5.7	1.6	2.6	< 0.0001

Composite end-point = in-hospital death, stroke and/or global brain ischemia. *MCS* Mechanical circulatory support, i.e., intraaortic balloon pump and/or extracorporeal membrane oxygenation. *P* values are adjusted for multiple covariates

### In-hospital mortality

Overall, 689 (17.7%) patients died during the hospital stay (Table 3). Logistic regression including multiple covariates, as above listed, provided probabilities for hospital mortality whose area under the receiver operating characteristic curve was 0.687 (95% CI 0.664–0.709). Logistic regression confirmed that four hospitals (I, N, O, P) were associated with increased risk of in-hospital mortality, while two hospitals (S, T) were associated with decreased risk of in-hospital mortality. The other factors independently associated with in-hospital mortality are summarized in Table 4. It is worth noting that in this regression model, operation performed by experienced surgeon was not a factor associated with decreased risk of in-hospital mortality (*p* = 0.646).

**Table 4:** Factors independently associated with in-hospital mortality

Covariates	Odds ratio (95% CI)	*P* value
Centers		
A	Reference	< 0.0001
B	0.830 (0.433–1.591)	0.575
C	1.106 (0.627–1.953)	0.728
D	1.725 (0.837–3.553)	0.139
E	0.962 (0.547–1.691)	0.892
F	0.838 (0.475–1.479)	0.542
G	1.232 (0.643–2.363)	0.529
H	0.666 (0.341–1.302)	0.235
I	2.140 (1.129–4.057)	0.020
L	0.768 (0.436–1.352)	0.360
M	0.818 (0.467–1.431)	0.481
N	2.305 (1.268–4.190)	0.006
O	1.855 (1.084–3.174)	0.024
P	2.269 (1.145–4.497)	0.019
Q	1.344 (0.800–2.259)	0.264
R	1.141 (0.598–2.180)	0.689
S	0.445 (0.224–0.885)	0.021
T	0.498 (0.266–0.933)	0.029
Age	1.036 (1.029–1.044)	0.000
Iatrogenic TAAD	2.178 (1.368–3.467)	0.001
Preoperative cardiac massage	5.045 (3.574–7.123)	0.000
Cerebral malperfusion	1.605 (1.306–1.972)	0.000
Mesenteric malperfusion	2.545 (1.758–3.684)	0.000
Peripheral malperfusion	1.840 (1.442–2.349)	0.000
Aortic root replacement	1.438 (1.167–1.771)	0.001
Total or partial aortic arch repair	1.665 (1.335–2.077)	0.000

*TAAD* type A aortic dissection

**Fig. 1:**
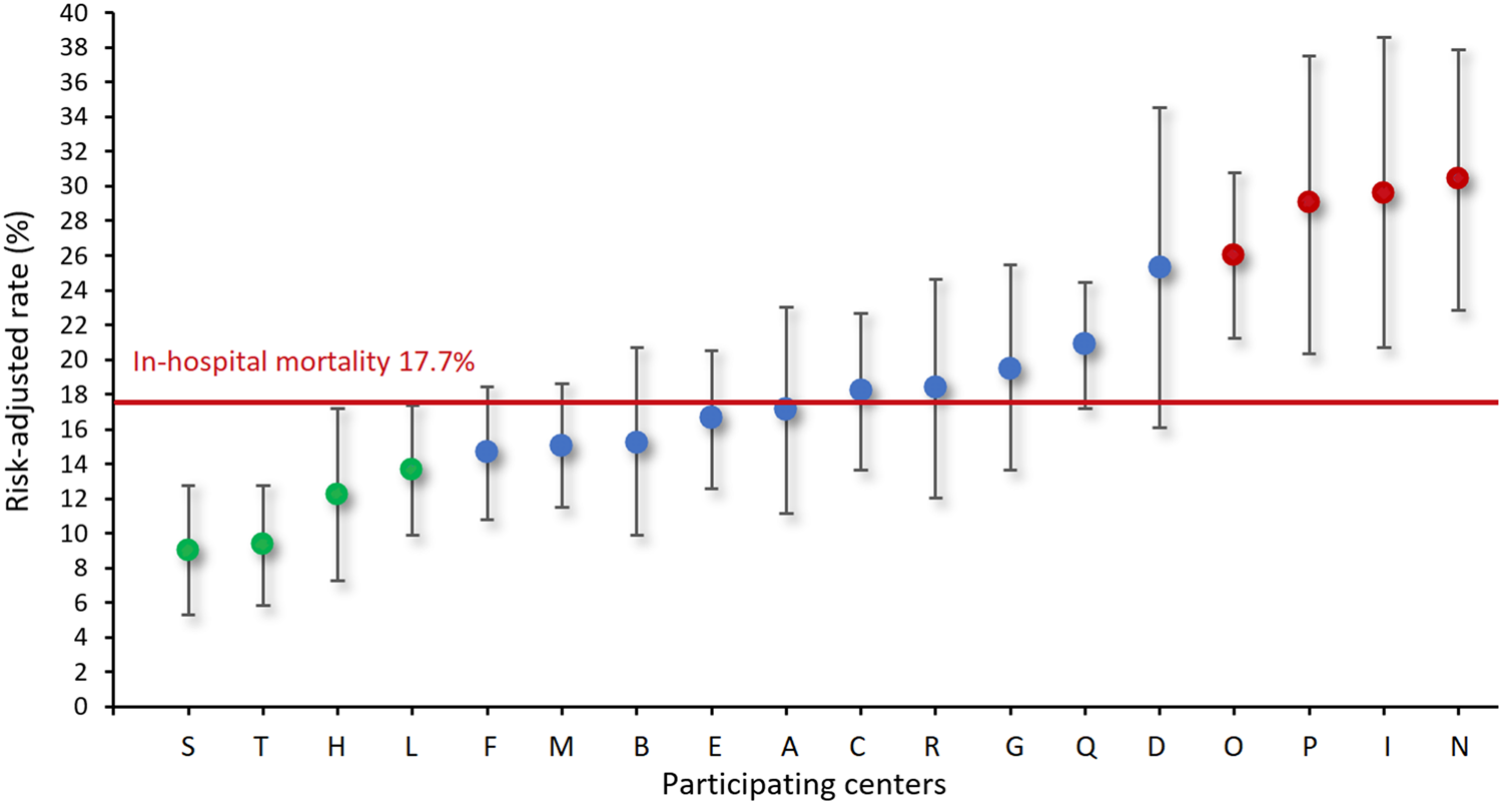
Risk-adjusted rates with 95% confidence intervals of in-hospital mortality after surgery for acute type A aortic dissection at the participating hospitals. Red line is the rate of in-hospital mortality in the overall series, red dots indicate hospitals with significantly higher risk-adjusted rates and green dots indicate hospitals with significantly lower risk-adjusted rates

Risk-adjusted in-hospital mortality rates of each participating hospital are shown in Fig. 1. Risk-adjusted in-hospital mortality rates were lower in four hospitals and higher in other four hospitals compared to the overall in-hospital mortality rate (17.7%) (Fig. 1).

### Propensity score matching analysis

**Fig. 2:**
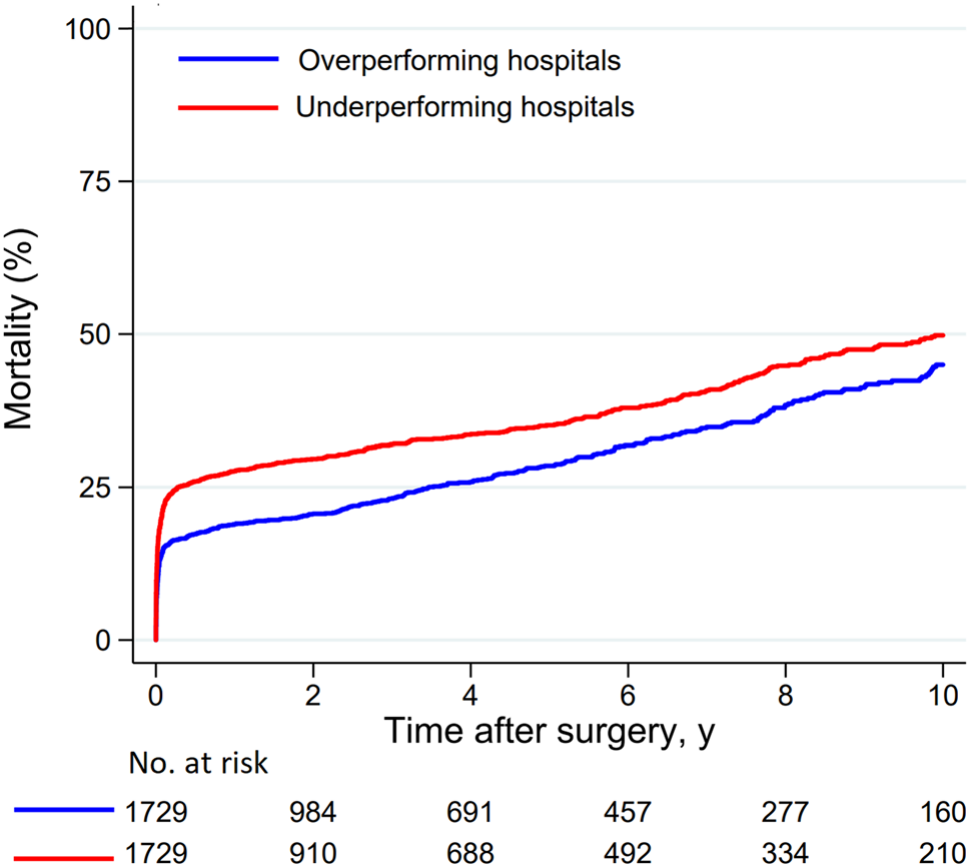
Long-term survival of propensity score matched patients operated for acute type aortic dissection at underperforming and overperforming hospitals

Participating hospitals were classified as overperforming or underperforming if their risk-adjusted in-hospital mortality rate was lower or higher than the in-hospital mortality rate of the overall series, respectively (Fig. 1). Based on this criterion, nine hospitals were classified as overperforming and nine hospitals as underperforming. Propensity score matching with a caliper of 1.4 yielded 1729 pairs with comparable baseline and operative covariates (Table 5). Among propensity score matched cohorts, overperforming hospitals had a significantly lower in-hospital mortality (12.8% vs. 22.2%, *p* < 0.0001) along with decreased rate of stroke and/or global brain ischemia (16.5% vs. 19.9%, *p* = 0.009) and mesenteric ischemia (2.5% vs. 5.0%, *p* < 0.0001) (Table 6). Overperforming hospitals had increased rate of re-exploration for intrathoracic bleeding (15.5% vs. 12.8%, *p* = 0.025). A 10-year, patients operated in overperforming hospitals had lower mortality (45.0% vs. 49.8%, *p* < 0.0001) (Fig. 2), but higher rate of distal aortic reoperation (9.8% vs. 5.2%, *p* < 0.0001). A trend toward increased risk of proximal aortic reoperation was observed in patients operated in overperforming hospitals (5.2% vs. 3.3%, *p* = 0.051).

**Table 5:** Patients’ characteristics and operative data of unmatched and propensity score matched cohorts

	Unmatched cohorts			Propensity score matched cohorts		
	Overperforming centers *N* = 2172	Underperforming centers *N* = 1730	Standardized differences	Overperforming centers *N* = 1729	Underperforming centers *N* = 1729	Standardized differences
Age, mean (SD), y	62.8 (13.0)	63.9 (12.9)	0.087	63.2 (13.0)	64.0 (12.9)	0.058
Females, No. (%)	674 (31.0)	511 (29.5)	0.033	534 (30.9)	510 (29.5)	0.030
Genetic syndrome, No. (%)	57 (2.6)	24 (1.4)	0.088	36 (2.1)	24 (1.4)	0.053
Bicuspid aortic valve, No. (%)	84 (3.9)	67 (3.9)	0.000	61 (3.5)	67 (3.9)	0.018
Iatrogenic dissection, No. (%)	59 (2.7)	44 (2.5)	0.011	47 (2.7)	44 (2.5)	0.011
Diabetes, No. (%)	102 (4.7)	94 (5.4)	0.033	84 (4.9)	94 (5.4)	0.026
Stroke, No. (%)	92 (4.2)	61 (3.5)	0.037	57 (3.3)	61 (3.5)	0.013
Pulmonary disease, No. (%)	213 (9.8)	114 (6.6)	0.117	136 (7.9)	114 (6.6)	0.049
Extracardiac arteriopathy, No. (%)	128 (5.9)	71 (4.1)	0.082	81 (4.7)	71 (4.1)	0.028
Prior cardiac surgery, No. (%)	72 (3.3)	50 (2.9)	0.025	58 (3.4)	50 (2.9)	0.027
Cardiac massage, No. (%)	91 (4.2)	76 (4.4)	0.010	73 (4.2)	76 (4.4)	0.009
Shock requiring inotropes, No. (%)	451 (20.8)	197 (11.4)	0.257	271 (15.7)	196 (11.3)	0.127
Cerebral malperfusion, No. (%)	496 (22.8)	333 (19.2)	0.088	362 (20.9)	333 (19.3)	0.042
Spinal malperfusion, No. (%)	52 (2.4)	30 (1.7)	0.046	38 (2.2)	30 (1.7)	0.033
Renal malperfusion, No. (%)	230 (10.6)	134 (7.7)	0.099	170 (9.8)	134 (7.8)	0.074
Mesenteric malperfusion, No. (%)	92 (4.2)	70 (4.0)	0.010	72 (4.2)	69 (4.0)	0.009
Peripheral malperfusion, No. (%)	371 (17.1)	172 (9.9)	0.210	224 (13.0)	171 (9.9)	0.096
DeBakey type I dissection, No. (%)	1814 (83.5)	1461 (84.5)	0.096	1435 (83.0)	1460 (84.4)	0.084
Operative data						
Experience aortic surgeon, No. (%)	1655 (76.2)	1574 (91.0)	0.407	1507 (87.2)	1573 (91.0)	0.122
Partial or total arch repair, No. (%)	525 (24.2)	251 (14.5)	0.247	355 (20.5)	251 (14.5)	0.159
Aortic root repair, No. (%)	680 (31.3)	417 (24.1)	0.161	496 (28.7)	417 (24.1)	0.104
XCT time, mean (SD), min	123 (61)	116 (56)	0.122	121 (60.8)	116 (56)	0.080
CPB time, mean (SD), min	224 (89)	206 (89)	0.207	220 (88)	206 (89)	0.155

*CPB* cardiopulmonary bypass, *SD* standard deviation, *XCT* aortic cross clamping time

**Table 6:** Outcomes in unmatched and propensity score matched cohorts

	Unmatched cohorts			Propensity score matched cohorts		
	Overperforming centers *N* = 2172	Underperforming centers *N* = 1730	*P* value	Overperforming centers *N* = 1729	Underperforming centers *N* = 1729	*P* value
Early outcomes						
Hospital mortality, No. (%)	305 (14.0)	384 (22.2)	< 0.0001	222 (12.8)	383 (22.2)	< 0.0001
Stroke/global brain ischemia, No. (%)	379 (17.5)	344 (19.9)	0.052	285 (16.5)	344 (19.9)	0.009
Composite end-point, No. (%)	581 (26.8)	571 (33.0)	< 0.0001	437 (25.3)	570 (33.0)	< 0.0001
Mesenteric ischemia, No. (%)	61 (2.8)	88 (5.1)	< 0.0001	43 (2.5)	87 (5.0)	< 0.0001
Dialysis, No. (%)	329 (15.2)	230 (13.3)	0.104	250 (14.5)	230 (13.3)	0.332
Reoperation for bleeding, No. (%)	327 (15.1)	222 (12.8)	0.047	268 (15.5)	222 (12.8)	0.025
MCS, No. (%)	71 (3.3)	70 (4.1)	0.0196	54 (3.1)	70 (4.0)	0.143
10-year outcomes						
Mortality, (%)	46.2	49.8	0.0002	45.0	49.8	< 0.0001
Distal aortic reoperation, (%)	9.5	5.2	< 0.0001	9.8	5.2	< 0.0001
Proximal aortic reoperation, (%)	5.2	3.3	0.040	5.2	3.3	0.051

Composite end-point = in-hospital death, stroke and/or global brain ischemia. *MCS* Mechanical circulatory support, i.e., intraaortic balloon pump and/or extracorporeal membrane oxygenation. *P* values are adjusted for multiple covariates

### Impact of hospital volume on in-hospital mortality

**Fig. 3:**
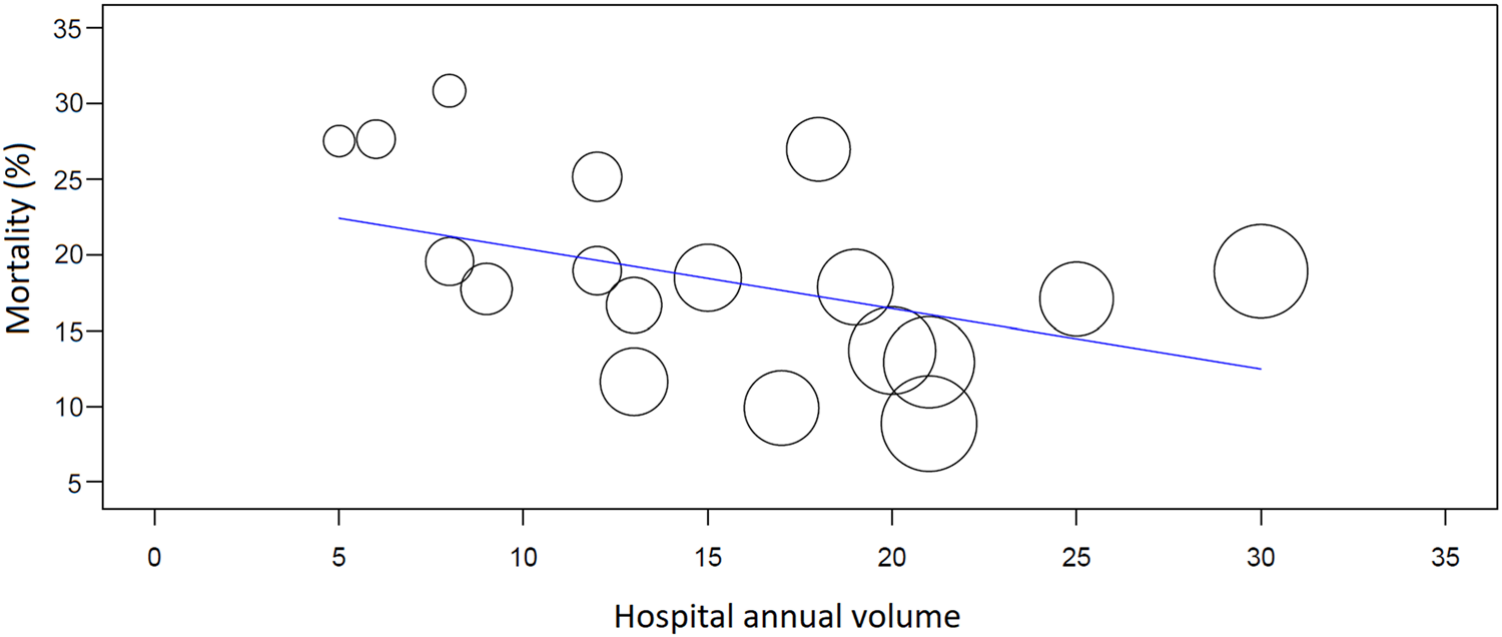
L’Abbé plot showing the effect of annual hospital volume of procedures for type A aortic dissection and the rate of in-hospital mortality (omnibus *p* = 0.043)

Aggregate meta-regression of the results of participating hospitals showed that hospital volume was inversely associated with in-hospital mortality (omnibus *p* = 0.043, intercept coefficient 0.244, 95% CI 0.177–0.311) (Fig. 3). The median hospital volume (14 cases/year) was considered as a cutoff for high-volume hospitals (9) and low-volume hospitals (9) and its effect was significant in aggregate data meta-regression. Furthermore, hospitals with an annual volume < 15 cases/year had an increased risk of in-hospital mortality in multivariable logistic regression (adjusted OR, 1.345, 95% CI 1.126–1.607).

## Discussion

The findings of the present study can be summarized as follows: (1) the early outcome of surgery for TAAD may significantly differ between hospitals; (2) low hospital volume of surgical procedure for TAAD may be a determinant of poor outcome.

The main aim of this study was to identify outlier hospitals in terms of perioperative mortality. We were able to identify four centers whose results seemed unsatisfactory compared to the other hospitals in terms of average in-hospital mortality. Furthermore, the estimation of risk-adjusted mortality rates allowed us to classify hospitals as under- or over-performing and propensity score matching prevented any imbalance in baseline and operative covariates inasmuch that these might potentially have affected the results. Indeed, we observed that overperforming hospitals performed more frequently aortic root and aortic arch replacement procedures and, despite this, their outcome was more favorable. Meta-regression showed that low volume of surgery for TAAD may explain such differences as it was confirmed by adjusted regression analysis. We observed that also 10-year mortality was lower in patients operated in overperforming hospital, but this seems mainly an effect of the initial lower early mortality. Overperforming hospitals had increased rates of late aortic reoperation. We hypothesize that these centers might have larger experience in aortic procedures, closer follow-up and had a more active policy of repair of aortic dissection-related complications also on the long run. However, the proportion of reoperation was rather low in both groups.

Regarding the impact of hospital volume on the outcome of surgery for TAAD, three large studies have previously investigated this issue. Kazui et al. [5] reported on a mortality of 16.3% in 10,097 TAAD patients operated on at 439 Japanese institutions from 2000 to 2004. In their series, only four hospitals had an annual volume of TAAD surgery of ≥ 20 cases and their mortality was 7.9%. Hospitals with annual volume < 5 cases had a mortality of 18.5% (OR 2.16, 95% CI 1.48–3.16). The difference between the high-volume and low-volume centers was statistically significant.

Brescia et al. [6] evaluated the outcome of 2918 patients who underwent surgery for TAAD patients operated at 232 hospitals from 2010 to 2014 in seven countries of the United States. The in-hospital mortality was 15.9% and was dependent on the hospital volume. The in-hospital mortality rate 20.8% in hospitals with of < 3 annual cases, 16.7% with 3–5 annual cases, 14.9% for 6–10 annual cases and 11.5% for more than 10 annual cases (*p* < 0.001).

Dobaria et al. [7] reported the results of 25,231 patients from the National Inpatient Sample (NIS) operated for TAAD between 2005 and 2014. Hospitals were classified as low-, medium- and high-volume based on tertiles of their open-thoracic aortic operative caseload and showed in hospital mortality of 21.5%, 16.8% and 11.6%, respectively (*p* < 0.001). Neurological complications were reported in 15.2%, 11.9% and 11.5%, respectively (*p* = 0.002). Importantly, the rates of overall complications were rather high, but they did not differ between the study groups (*p* = 0.11). This study provided the benefits of analyzing their results based on the aortic surgery caseload rather than the TAAD caseload. The former caseload may be a more reliable measure of the surgical and anesthesiological expertise than the less frequently encountered TAAD. Interestingly, the overall complication rates were similar between the study groups, still in-hospital mortality was significantly lower among high-volume hospitals. Such findings were observed also in the propensity score matched cohorts of this study. These findings suggest that, despite a high incidence of postoperative complications, overperforming hospitals can provide a better perioperative care for these complications than underperforming centers.

Consonant with these results, Mariscalco et al. [3] reported the results of a pooled analysis on 30 studies on acute aortic syndrome and showed that high-volume centers were associated with significantly lower mortality (OR 0.51, 95% CI 0.46–0.56). These authors demonstrated also that high-volume surgeons decreased the risk of mortality as well (OR 0.41, 95% CI 0.25–0.66). The findings are similar to those observed after surgery for ruptured abdominal aortic aneurysm, in which hospital volume, but not surgeon’s volume, had a significant impact on perioperative mortality [8].

The retrospective nature is the main limitation of this study. Second, we do not have data on individual surgeon’s volume. Despite the relatively large size of this database, this would not have allowed a reliable analysis of this important issue because the number of procedures for TAAD per surgeons might still have been rather small. Third, no data were available on the proportion and outcome of patients who were not treated for aortic-related complications. Finally, participating hospitals might have differed significantly for referral pathway and distance from patient’s residence. This means that hospitals might have a lower caseload because referral of patients is slower as several patients might have died during transportation. Similarly, long distances may be associated with worse clinical conditions, which cannot be stratified only by clinical variables.

In conclusion, the present findings indicate that there are significant differences between hospitals in terms of early outcome after surgery for TAAD. Low hospital volume of TAAD may be a determinant of poor outcome in these patients.
